# Impact of aging and body mass index on upper extremity motor unit number index and size

**DOI:** 10.3389/ebm.2025.10491

**Published:** 2025-07-24

**Authors:** Lauren I. Gulley Cox, Nicholas Dias, Chuan Zhang, Yingchun Zhang, Stacey L. Gorniak

**Affiliations:** ^1^ Department of Health and Human Performance, University of Houston, Houston, TX, United States; ^2^ Department of Biomedical Engineering, University of Houston, Houston, TX, United States

**Keywords:** motor unit loss, adiposity, neuromuscular, MUNIX, MUSIX

## Abstract

The focus of this study was to evaluate motor unit number and size across the upper extremity in older adults (aged 60+ years) versus young healthy adults (aged 20–30 years). We hypothesized that older adults would have: fewer motor units and increased motor unit size as compared to young healthy adults (H1), that motor unit size would differ across the upper extremity muscles as compared to young healthy adults (H2), and higher body mass index (BMI) would be associated with lower motor unit numbers (H3). Compound muscle action potential (CMAP), motor unit number index (MUNIX), and motor unit size index (MUSIX) were evaluated in five muscles of the upper extremity. Group differences in CMAP due to aging were accounted for by increased body mass index (BMI); group differences in MUSIX were not impacted by BMI. No difference in MUNIX was found; however, an influence of BMI was found across groups. While this data provides supporting evidence of age-related motor unit changes, body composition changes with age may confound these conclusions when surface electromyography is utilized as the measurement modality. Adiposity estimation should be considered in future EMG studies, particularly in populations with higher BMI values.

## Graphical Abstract

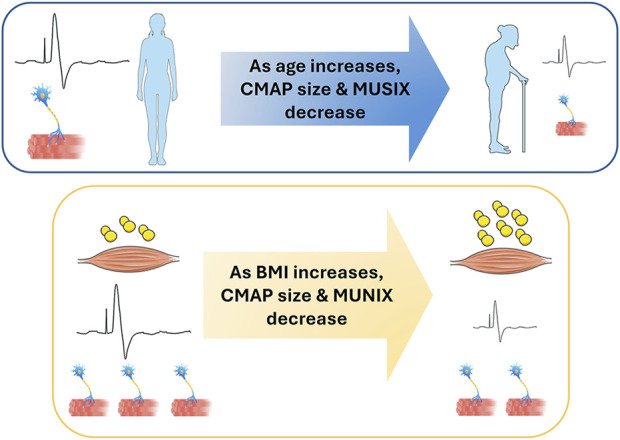

## Impact statement

The data in this project indicate significant changes in motor unit characteristics of the upper extremity in older adults that may be attributable to increased body mass index (BMI) with aging.

## Introduction

Motor unit loss is a key characteristic of neuromuscular diseases and motor deficits that are associated with aging [1–5]. At the muscle level, aging is associated with delayed muscle activation in response to stimulus along with altered muscle recruitment—an indicator of neuromuscular dysfunction [6]. Despite this, the literature on age-associated neuromuscular dysfunction is inconsistent with data suggesting that muscular alterations are dependent on muscle location and muscle function [2, 7–9].

Work by Dalton et al. [8] did not report differences in motor unit number estimates in select lower extremity muscles between older adults (aged 70+ years) and young healthy adults (aged 20–30 years), concluding there to be no age associated reductions in motor unit number estimates in the lower extremities. Conversely, age associated motor unit loss in muscles of the upper extremity has been reported. Specifically, Brown et al. [7] found subjects over the age of 60 had one half the number of motor units in the biceps brachii as compared to those under 60 years of age. Further investigation by Galea et al. [2] into the number of motor units within the biceps brachii, extensor digitorum brevis, and median innervated thenar muscles did not arrive at the same conclusion, despite reporting diminished peak-to-peak amplitude and reduced area of the maximum M-wave in muscles of the upper extremity with advanced age. Instead, findings from Galea et al. [2] indicate reduced motor unit numbers in distal muscles of the upper extremity; however, supporting data for this conjecture are sparse.

Additional work has shown higher motor unit discharge rates in younger adults via meta-analysis of data collected from the lower extremity; however, variability in methods and muscles assessed in the meta-analysis limits age-related interpretations for the upper extremities [10] in studies that included various upper extremity muscles such as biceps brachii, triceps brachii, abductor digiti minimi, first dorsal interosseus, and extensor digitorum. In contrast, other work indicates more homogeneity of motor unit output primarily in the lower extremities with aging [11]. Taken together, these findings indicate that natural aging affects the neuromuscular system may manifest differently in the upper and lower extremities. These disparities highlight the need for further investigation.

In addition, it is well recognized that body mass index increases (BMI) with age—nearly 1/3 of the US population aged 60+ years meets the BMI criteria for obesity [12]. Increased adiposity (and increased BMI) has been noted as a barrier to muscle tissue assessment via surface electromyography (EMG) [13–15]. It is unclear if increased BMI with age also impacts measured motor unit numbers and estimated motor unit sizes. This inclusion is pertinent given that increased BMI, a common occurrence with aging, could potentially confound the assessment of motor units, blurring the distinction between age-related changes and those due to increased adiposity.

In light of these gaps, the focus of the current study was to evaluate motor unit number and size across the upper extremity in older adults (aged 60+ years) versus young healthy adults (aged 20–30 years). We hypothesized that older adults would have fewer motor units and increased motor unit size in muscles of the upper extremity as compared to young healthy adults (Hypothesis 1), and that motor unit number and size would differ across the upper extremity muscles examined in older adults as compared to young healthy adults (Hypothesis 2). We also hypothesized that BMI would have an impact on CMAP values and motor unit numbers, such that higher BMI values would be associated with lower CMAP values and motor unit numbers, particularly in older adults—as older adults generally exhibit higher BMIs (Hypothesis 3).

## Materials and methods

### Participants

Thirteen (13) young healthy controls (5M, 8F) and 12 older adult participants (6M, 6F) were recruited for this study from the greater Houston area (population approx. 2.3 million), see Table 1 for demographics. All participants were right-handed (laterality quotient (LQ) >40, assessed with the Edinburgh Handedness Inventory). Exclusion criteria for both groups included: diagnosis of Type 1 or Type 2 Diabetes, history of uncontrolled hypertension, history of limb amputation, chemotherapy, or neurological diseases (Alzheimer’ Disease, Dementia, Huntington’s Disease, Traumatic Brain Injury, Multiple Sclerosis, Parkinson’s Disease, Paraproteinemic Demyelinating Neuropathy (PDN), Muscular Dystrophy, Carpal Tunnel Syndrome, Charcot-Marie-Tooth Disorder, and any other neuropathies), and pain in the extremities that limits activities of daily living. This study was approved by the Institutional Review Board (IRB) at the University of Houston in accordance with Declaration of Helsinki. All subjects provided written informed consent.

**TABLE 1:** Study participant characteristics.

Characteristic	Young	Older adult
N (Males, Females)	13 (6, 7)	12 (6, 6)
Age (y)	24.5 ± 4.0	68.1 ± 4.5
Height (m)	1.635 ± 0.161	1.717 ± 0.099
Mass (kg)	65.07 ± 18.8	89.4 ± 28.7
BMI (kg/m^2^)	23.8 ± 3.0	30.3 ± 11.3
LQ	80 ± 18	92 ± 12

Values are mean ± SD or count. BMI, body mass index; LQ, laterality quotient.

### EMG recording

Multichannel surface EMG was recorded from each muscle using a bioamplifier (FE234 Quad BioAmp, ADInstruments, Colorado Springs, CO, USA) and PowerLab data acquisition system (PowerLab 8/35, ADinstruments, Colorado Springs, CO, USA). Prior to attaching surface electrodes (3M Red Dot 2560 Foam Monitoring Electrodes with Sticky Gel, 3M, Saint Paul, MN, USA), the skin was cleaned with alcohol. Surface electrodes used consisted of diaphoretic solid gel in disc shape; gel disc diameter was 18 mm, size of the electrode was 25 mm × 27 mm inclusive of foam adhesive materials. The longitudinal axis of each of the muscles (abductor pollicis brevis (APB), biceps brachii (BB), extensor digitorum (EDC), flexor digitorum superficialis (FDS), and triceps brachii (TRI)) was identified via palpation. Placement was based on [16]. Two surface electrodes were placed on the muscle belly, along the longitudinal axis of the respective muscle; center-to-center interelectrode distance was 26–30 mm. A reference electrode was placed on a bony process located proximally to the muscle being tested while a ground electrode was placed distally. EMG data was acquired continuously at 1,000 Hz using LabChart software (ADInstruments, Colorado Springs, CO, USA). Any channel crosstalk was inspected manually and electrodes were repositioned if evidence of channel crosstalk was present.

### Nerve stimulation and CMAP

Maximum compound muscle action potential (CMAP), a measure that describes the maximal electrophysiological size of the entire motor pool within a muscle, was obtained for each muscle by supra-maximal stimulation of the innervating nerve (APB: median; BB: musculocutaneous; EDC: radial; FDS: median; TRI: radial), with a DS7A muscle current stimulator (Digitimer, United Kingdom). Stimulation intensity generally started around 5–30 mA and was increased in increments of approximately 20% until a maximal response was reached. The duration for a single pulse stimulation was 200 µs. The nerve was then stimulated with 120% of the final intensity to confirm the maximum CMAP was reached and confirmed visually, consistent with [17]. Major differences in the approach employed in the generation of this data set as compared to [18] include the use of 300 ms epochs and standardized electrode placement, as per [16]. The use of 300 ms epochs meets the minimum epoch duration as per [18] to identify tremor, but not the recommended 500 ms. Our data was collected using a standardized electrode placement to ensure reproducibility of the data, in contrast to “electrode placement for CMAP optimization” as endorsed in [18].

### Isometric contractions, MUNIX, and MUSIX

For all tasks, participants were seated in a chair facing the testing table with his/her upper arms at approximately 20° of abduction in the frontal plane. The forearm of each participant rested on a padded surface with an elbow angle of approximately 135° in the sagittal plane. The wrist orientation was such that the hand was restrained in a neutral position (neutral flexion/extension, neutral radial/ulnar deviation) during testing. Participants performed isometric contractions via an externally fixed load cell (Model SM-500, Interface Force Measurement Solutions, Scottsdale, AZ, USA) with the hand in: pronation to evaluate TRI and EDC, and in supination to evaluate BB, FDS, and ABP. Directionally of the load cell was modified to accommodate force production during testing. Participants performed three maximum voluntary contraction (MVC) trials of 10–15 each, with one minute of rest between trials. The highest MVC force was used to determine the target forces for the submaximal contraction trials. After MVC trials, participants were asked to perform 30-second submaximal isometric contractions each at 5, 15, 25, 50, and 75% MVC. Force produced by the subject was used as visual feedback to maintain the contraction level. This testing procedure was performed for all five muscles (APB, BB, EDC, FDS, and TRI).

The surface EMG interference pattern was recorded throughout each contraction at varying levels of force. Motor unit number index (MUNIX), an electrophysiological measure of the number of motor units within a muscle that is easy to perform and well tolerated by study participants, was used to estimate the number of motor units contained in each muscle using maximum CMAP produced during voluntary isometric muscle contractions in 300 ms epochs. Additionally, motor unit size was estimated by calculating the motor unit size index (MUSIX), an indicator of the size of motor units within the evaluated motor pool within a muscle, which is derived using MUNIX and CMAP values. Data underwent bandpass filtering (10–450 Hz) prior to analysis. Additional details on how to calculate CMAP, MUNIX, and MUSIX can be found in [19].

### Statistical analysis

SPSS version 30.0 (SPSS IBM, New York, NY, USA) was used to perform parametric statistical analyses. Outliers were identified in SPPS using Tukey’s method while creating initial boxplots of data. The following # of outliers were removed from the data set as indicated via Tukey’s method: CMAP (1 young), MUNIX (2 young, 2 older), MUSIX (5 young, 4 older). For each variable of interest, automatic linear modeling (ALM) was used to select significant covariates (specifically age and BMI) using forward stepwise selection in SPSS [20]. Follow-up correlation analyses were performed for all ALM-identified significant covariates. Data were analyzed using two-way ANCOVAs to compare between *Groups* (T2D and Control). Within-subject factors for neuromuscular evaluation was *Muscle* (APB, BB, EDC, FDS, and TRI) as was a *Group x Muscle* interaction. Regression models between measures of interest (CMAP, MUNIX, and MUSIX) and BMI were calculated in OriginLab 2025 (Northampton, MA, USA) for each group separately.

## Results

### CMAP

**FIGURE 1:**
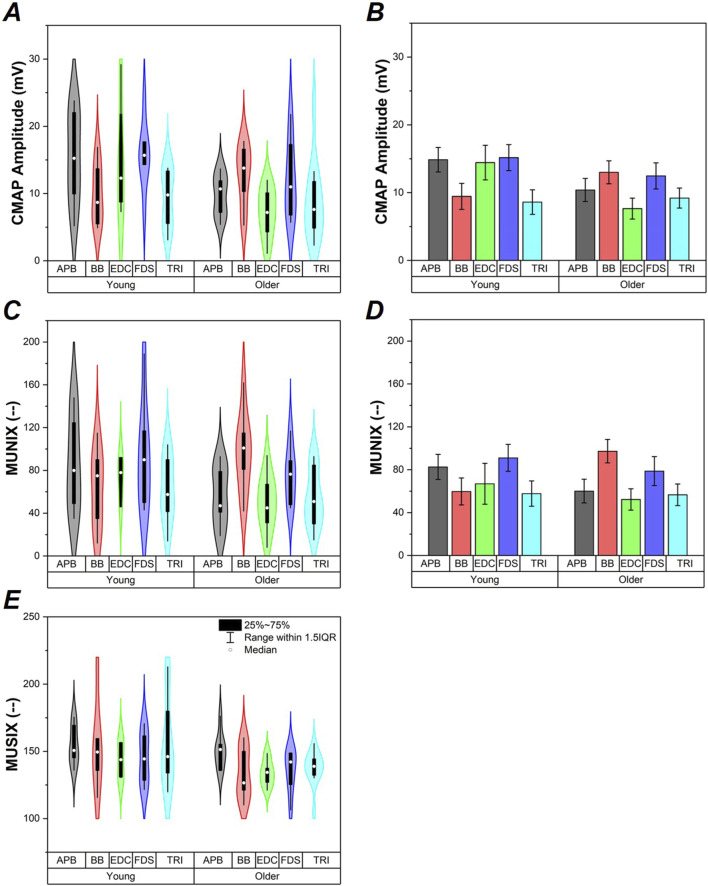
CMAP, MUNIX, and MUSIX values (**A-C** respectively). Data from study participants are shown via violin plots in panels **(A,C,E)**. Average ±SE values of CMAP and MUNIX data generated as a result of ANCOVA models shown for reference to clarify *Group* effects in panels **(B,D)**. Data are shown for the older adult and young groups, as well as for each individual muscle (APB, BB, EDC, FDS, and TRI).

A baseline two-way ANOVA was performed for maximum CMAP amplitude with *Group* (Young and Older Adult) and *Muscle* (APB, BB, EDC, FDS, and TRI) as main factors. *Group* (F_1,72_ = 6.300, *p* < 0.05) was found to be significantly different such that the young adult group had significantly larger overall max CMAP amplitude compared to older adults, Figure 1A. No significant main effect of *Muscle* nor *Group x Muscle* interaction were found.

**FIGURE 2:**
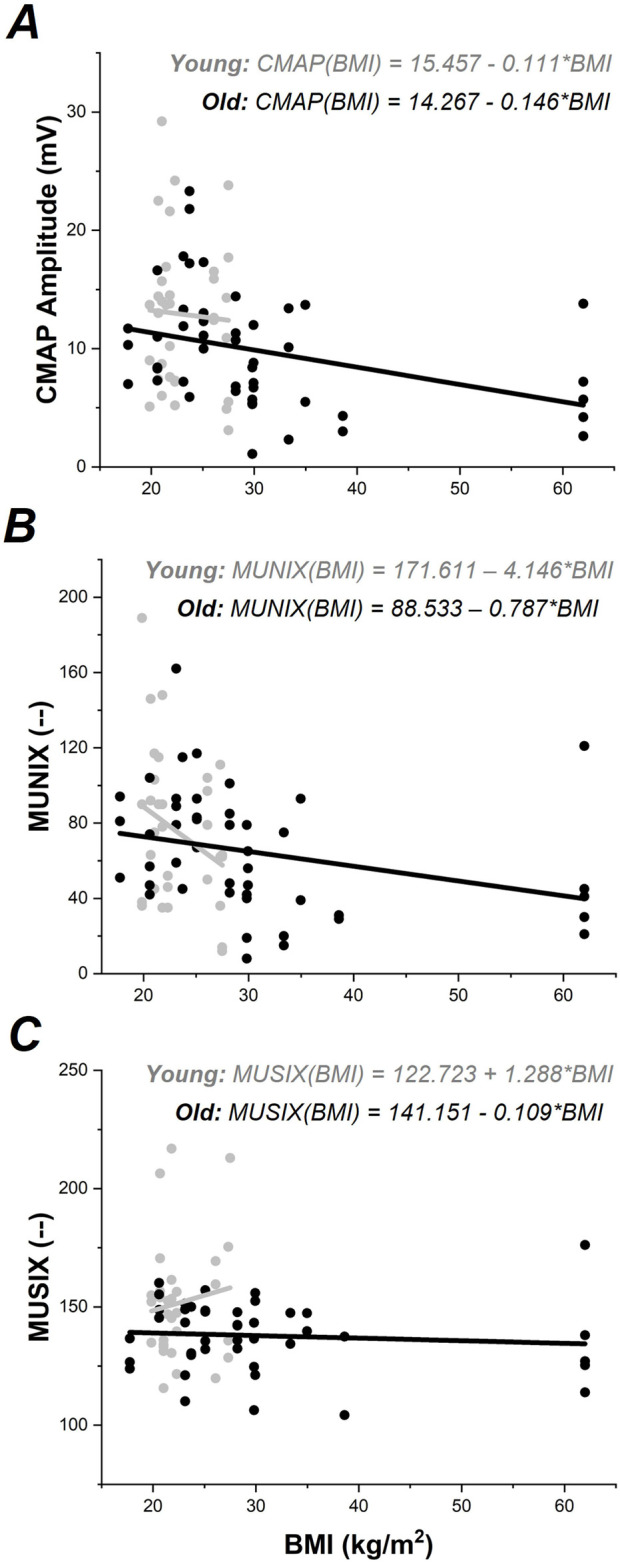
CMAP, MUNIX, and MUSIX values plotted against BMI for each group. Young data shown in gray; Older Adult data shown in black. Regression models for each group are shown separately according to color.

ALM modeling indicated *BMI* as a covariate in the CMAP amplitude model which replaced the *Group* effect, Figure 1B. Follow-up ANCOVA was performed with *Group* and *Muscle* as main factors and *BMI* as a covariate. Only *BMI* (F_1,71_ = 5.439, *p* < 0.05) was found to be significant. Increased *BMI* was associated with reduced max CMAP amplitude (r_79_ = −0.245, p < 0.05). Regression models calculated for each group separately did not show a significant relationship between CMAP and BMI in the Young group (p >0.7); however, a significant relationship between CMAP and BMI emerged in the Older Adult group (F_1,46_ = 6.62, p < 0.05), shown in Figure 2A.

### MUNIX

A baseline two-way ANCOVA was performed for MUNIX with *Group* (T2D and Control) and *Muscle* (APB, BB, EDC, FDS, and TRI) as main factors. No main effects or interactions were found, Figure 1C.

ALM modeling indicated *BMI* as a covariate in the MUNIX data, Figure 1D. Follow-up ANCOVA was performed with *Group* and *Muscle* as main factors and *BMI* as a covariate. Only *BMI* (F_1,68_ = 4.701, *p* < 0.05) was found to be significant. Increased *BMI* was associated with reduced MUNIX (r_76_ = −0.243, p < 0.05). Regression models calculated for each group separately indicated a nearly significant relationship between MUNIX and BMI in the Young group (F_1,31_ = 3.03, p = 0.09) and a significant relationship between MUNIX and BMI in the Older Adult group (F_1,44_ = 4.19, p < 0.05), shown in Figure 2B.

### MUSIX

A baseline two-way ANCOVA was performed for MUSIX with *Group* (T2D and Control) and *Muscle* (APB, BB, EDC, FDS, and TRI) as main factors. *Group* (F_1,64_ = 6.633, *p* < 0.05) was found to be significantly different such that the young adult group had significantly larger MUSIX compared to older adults, Figure 1E. No significant main effect of *Muscle* nor *Group x Muscle* interaction were found.

ALM modeling did not indicate any significant covariates in the MUSIX data, thus follow-up ANCOVAs were not performed. Regression models calculated for each group separately did not show significant relationships between MUSIX and BMI (p > 0.5), Figure 2C.

## Discussion

The purpose of this study was to evaluate motor unit characteristics across the upper extremity in older adults (aged 60+ years) as compared to young healthy adults (aged 20–30 years). We hypothesized that older adults would have fewer motor units and increased motor unit size in muscles of the upper extremity as compared to young healthy adults (Hypothesis 1). Overall, this hypothesis was not supported as no age-related changes in MUNIX were found concurrent despite evidence of lower MUSIX in older adults. We also hypothesized that motor unit number and size would differ across the upper extremity muscles examined in older adults as compared to young healthy adults (Hypothesis 2). This hypothesis was also not supported, as no main effects of *Muscle* were found. In Hypothesis 3, we explored the impact of BMI on CMAP, MUNIX, and MUSIX measures. BMI replaced the age group effect in our CMAP results, was a significant factor in the MUNIX results, but did not impact MUSIX results. The implications of these findings are discussed in the following paragraphs.

The data produced in this study do not support the conjecture that aging is associated with an overall reduction of motor units in the upper extremity. While age-related changes in CMAP values were found between age groups, this difference was accounted for by group differences in BMI—indicating that BMI is an important confounder of electrophysiological activity across the lifespan. This is consistent with emerging reports of increased adiposity as a significant barrier to EMG measurement [13–15, 21, 22], providing a physiological basis for this reported effect. Recent work has indicated that higher amounts of adiposity (generally assessed via BMI) are associated with reduced amplitude of electromyographic (EMG) signals [13–15]. The increased BMI in the older adult group in this study reflects an increase in body mass with aging, as evidenced by Table 1 and Figure 2. This increase in body mass is likely due to increased adiposity concurrent with sarcopenia across the body with age [23]. Increased adiposity with advanced age is preceded by a metabolic cascade, including reduced blood glucose and triglyceride clearance—leading to increased fat deposition within the body as age increases [24, 25]. Increased adiposity results in a thicker insulation layer between the derma and muscle that reduces the strength of EMG signal measured. This in turn reduces the CMAP amplitude measured and impacts any measurements that depend on CMAP measured from surface EMG for computation (e.g., MUNIX and MUSIX). While MUNIX was impacted by BMI in this data set, MUSIX was not—despite a finding of reduced MUSIX values in the older adult group. This is a highly relevant finding, as loss of motor unit number with aging is assumed to precede sarcopenia with advanced age [26]. The data in this paper suggest that the loss of motor unit number may be concurrent with sarcopenia in the upper extremities in older adults.

In order to take into account adiposity-associated EMG signal attenuation in motor unit characterization, adiposity measurement (via dual-energy x-ray absorptiometry (DXA)) or adiposity estimation via BMI should be considered and controlled for statistically in future work, particularly in populations living with higher BMI values due to chronic disease (e.g., cardiovascular disease, stroke, etc.). Use of either DXA or BMI is warranted as attempts to use skinfold thickness to estimate adiposity have been found to be inaccurate, particularly in persons with BMI >30 kg/m [2, 27, 28]; whereas use of either DXA of BMI has produced consistent results with respect to each other in terms of accounting for adiposity impacts in evaluation of neurological and muscular measurements [21, 22, 29–32]. One way that adiposity as a confound may be accounted for in future work is by creating mathematical or statistical models of adiposity distribution [21, 22] that could be employed in signal processing of EMG data. This approach may aid in distinguishing physiological changes in neuromuscular function from those induced by signal attenuation during EMG measurement due to increased adiposity.

In addition to these findings, none of the measures considered in this project were significantly different based on muscle location. These data are in contrast to work by Galea et al. [2], in which differences in motor unit measures of the distal musculature of the upper extremity (ABP, EDC, FDS) would have been reduced in older adults as compared to motor unit measures in more proximal musculature of the upper extremity (BB). These data support prior findings of [11] in terms of homogeneity of motor unit measures within a limb with aging, in contrast to reports of motor unit loss in the distal musculature with age [2, 33–35]. More work is needed to clarify the physiological mechanisms responsible for distal motor unit changes with age [25]—particularly with the consideration of increased adiposity with age functioning as a confounding factor in EMG measurement.

## Conclusion

The data produced in this study do not support the conjecture that aging is associated with a reduction of motor unit number index in the upper extremity; however, evidence to support age-related changes in motor unit size was found. Group differences in CMAP values due to aging were accounted for by increased BMI. The data do not support reports of motor unit loss in distal musculature with age. Adiposity estimation via BMI should be considered and controlled for statistically in future work, particularly in populations living with higher BMI values.

## Data Availability

Data analyzed in this project is available from https://doi.org/10.5281/zenodo.15864514.
